# A critical review on polydopamine surface-modified scaffolds in musculoskeletal regeneration

**DOI:** 10.3389/fbioe.2022.1008360

**Published:** 2022-11-18

**Authors:** Hamidreza Tolabi, Negar Bakhtiary, Shaghayegh Sayadi, Maryam Tamaddon, Farnaz Ghorbani, Aldo R. Boccaccini, Chaozong Liu

**Affiliations:** ^1^ New Technologies Research Center (NTRC), Amirkabir University of Technology, Tehran, Iran; ^2^ Department of Biomedical Engineering, Amirkabir University of Technology (Tehran Polytechnic), Tehran, Iran; ^3^ Burn Research Center, Iran University of Medical Sciences, Tehran, Iran; ^4^ Department of Biomaterials, Faculty of Interdisciplinary Science and Technology, Tarbiat Modares University, Tehran, Iran; ^5^ School of Mechanical Engineering, College of Engineering, University of Tehran, Tehran, Iran; ^6^ Institute of Orthopaedic and Musculoskeletal Science, University College London, Royal National Orthopaedic Hospital, Stanmore, United Kingdom; ^7^ Institute of Biomaterials, Department of Materials Science and Engineering, University of Erlangen-Nuremberg, Erlangen, Germany

**Keywords:** polydopamine, surface engineering, tissue engineering, musculoskeletal tissue, scaffolds

## Abstract

Increasing concern about age-related diseases, particularly musculoskeletal injuries and orthopedic conditions, highlights the need for strategies such as tissue engineering to address them. Surface modification has been developed to create pro-healing interfaces, personalize scaffolds and provide novel medicines. Polydopamine, a mussel-inspired adhesive polymer with highly reactive functional groups that adhere to nearly all substrates, has gained attention in surface modification strategies for biomaterials. Polydopamine was primarily developed to modify surfaces, but its effectiveness has opened up promising approaches for further applications in bioengineering as carriers and nanoparticles. This review focuses on the recent discoveries of the role of polydopamine as a surface coating material, with focus on the properties that make it suitable for tackling musculoskeletal disorders. We report the evolution of using it in research, and discuss papers involving the progress of this field. The current research on the role of polydopamine in bone, cartilage, muscle, nerve, and tendon regeneration is discussed, thus giving comprehensive overview about the function of polydopamine both *in-vitro* and *in-vivo*. Finally, the report concludes presenting the critical challenges that must be addressed for the clinical translation of this biomaterial while exploring future perspectives and research opportunities in this area.

## Introduction

Musculoskeletal injuries may affect any of the following tissue types: bone, cartilage, nerve, tendon, and muscle, and treatment will depend on the severity of the injury (Zhao et al., 2019; Naghieh et al., 2021). Over 1.71 billion people worldwide have musculoskeletal disorders (WHO, 2021), leading to an exponential demand for tissue engineering (TE) studies to help regenerate injured tissues. The treatment outcome of musculoskeletal disorders is affected by mechanical properties, bioactivity, immune response, and cytotoxicity of scaffolds, which immensely act tissue reconstruction and the success of the implantation process (Bakhtiary et al., 2021; Heid et al., 2022).

Biomaterials-tissue contact interface of scaffolds which are an integral part of a regeneration process induces immune reactions in the patient’s body (Arab-Ahmadi et al., 2021). Moreover, tissue regeneration is affected by the secretion of immune cells during tissue formation, and in case of any abnormal reaction, scaffolds are prone to failure. However, tissue repair can be induced by the immune system and significantly enhanced according to the condition (Yang et al., 2019). For instance, by altering the implant surface, tissue integration and antimicrobial properties can be highly enhanced (Gao et al., 2017a). Hence, the surface properties of scaffolds are critical factors that must be considered since they affect cell attachment and proliferation (Gao et al., 2017b; Alfieri et al., 2022).

Over the last decade, polydopamine (PDA), a mussel-inspired adhesive polymer with a highly reactive functional group including amine, imine, and catechol that adheres to nearly all different substrates and immobilizes biomolecules, has been increasingly investigated. A variety of cellular responses, such as cell spreading, proliferation, migration, and differentiation, can also be influenced by it in the body, enhancing *in-vivo* performance of scaffolds (Jia et al., 2019; Rezaei et al., 2021). PDA may properly functionalize a broader range of surfaces than other surface modification materials (Ghorbani et al., 2019a; Cheng et al., 2019). PDA presented high biocompatibility by simulating cell interaction as a newly found surface modification material. As a result, it has gained attention and has been widely used in various areas over the last decades (Wang et al., 2015; Ryu et al., 2018; Ghorbani et al., 2020a). This review aims to investigate PDA influence as a coating material in TE, study the evolution of using PDA in scaffold coating and report the recent developments of PDA surface-modified scaffolds in musculoskeletal TE.

## Polydopamine as a coating material for enhancing surface functionality of biomedical devices

**FIGURE 1:**
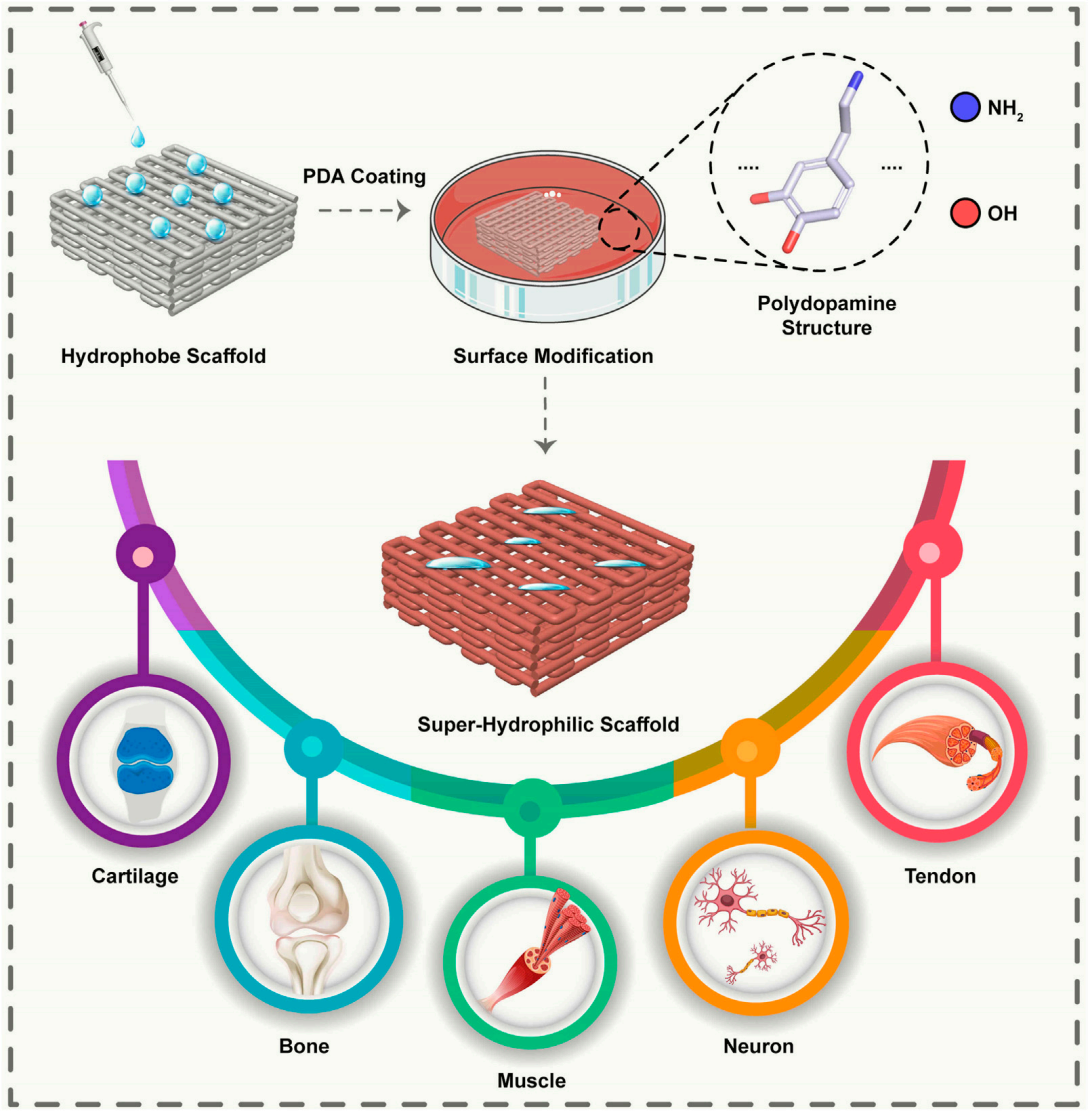
Schematic representation of a surface modified with PDA leads to a super hydrophilic surface and enables the adhesion of cells to the surface. It is possible to use scaffolds modified with PDA for various applications in various musculoskeletal tissues.

PDA, as a synthetic eumelanin polymer derived from dopamine (DA), has a molecular structure similar to 3,4-dihydroxy-l-phenylalanine, consists of oligomers that differ in chain lengths (trimers and tetramers are abundant) and different monomers compositions (Liebscher, 2019; Davari et al., 2022). PDA is characteristically exceptional, extremely biocompatible, and has a distinctive chemical structure of a different functional group that includes catechol, amine, and imine, helping to immobilize molecules and absorb metal ions. Hence has been widely used in regulating tissue and cellular response to materials (Shokrollahi et al., 2020; Ghorbani et al., 2021). PDA’s adhesive intensity is attributed to its catechol and aminoethyl groups (Gao et al., 2019; Sarkari et al., 2022). A reduction in aminoethyl groups through extensive 5,6-dihydroxyindole (DHI) synthesis reduces adhesiveness. Therefore, PDA coatings endow the surface with hydrophilic properties **(** Figure 1 **)** (Liebscher, 2019). PDA has a high level of cellular affinity and the capability to reduce inflammation, immunological response, and cytotoxicity (Hong et al., 2011; Han et al., 2017; Singh et al., 2021).

PDA coatings possess many advantages compared to conventional surface modification strategies, and recently PDA coating mechanisms, chemical structure, and potential applications have been extensively discussed (Ryu et al., 2018; Barros et al., 2021). The PDA coating procedure consists of two steps. The first phase includes the DA monomers autooxidation in which DA quinone is formed in the existence of dissolved oxygen, alkaline buffer, and a sufficient amount of DA hydrochloride monomers, leading to the formation of PDA. The crucial step in PDA constitution is the DA oxidation to dopaquinone in aerobic conditions (van der Leeden, 2005; Jia et al., 2019). Despite the existence of two models: “covalent bonding” or “noncovalent bonding”, the second phase of PDA formation remains elusive. Although the adhesion processes are unknown yet, they are linked to chemical compositions and activity. Since covalent and non-covalent bindings are the two basic types of adhesion mechanisms, an increase in non-covalent bonds will result in stronger adhesion (Gao et al., 2019).

The polymerization process and the mechanism of dopamine formation on different surfaces are similar. Still, some parameters of PDA coating formation, such as immersion time and growth rate and PDA layer formation on different surfaces, are different, as well as some results, such as the thickness of the formed layer and the homogeneity of the layer can be varied depend on the surface (Kang et al., 2009; Lynge et al., 2011; Cui et al., 2018; Ryu et al., 2018).

PDA coatings exhibit several desirable characteristics, including non-specific deposition, processing under mild and controlled conditions to prevent damage during the irradiation process, and preserving scaffolds’ permeability (Miller et al., 2017). In this process, PDA acts as either an oxidant or a reductant which is possible due to its oxidizing quinonyl and reductive catechol. Whatever the surface’s hydrophilicity or hydrophobicity, PDA forms on organic or inorganic surfaces, affecting the surface characteristic of composite materials (Ryu et al., 2018). In addition, because PDA rarely binds to chemicals other than water and solutions containing metal ions, it is helpful in the deposition (Miller et al., 2017). During the coating process, surfaces are immersed in an aqueous alkaline DA hydrochloride solution over a period of time, and following bonding, PDA serves as a secondary platform for functional materials to bind to the surface. (Miller et al., 2017; Ryu et al., 2018). The thickness of PDA coating depends on many factors, including polymerization time, pH, solvents, dopamine concentration, and most importantly, the type of substrate and the temperature. For instance, as a result of using Gompertz’ equation and data reported in the research, Habibi Rad et al. (Habibi Rad et al., 2018) presented a kinetics model for PDA layer growth based on the results obtained on different substrates and temperatures. Based upon the reported data, Gompertz’ equation was chosen, which indicates that the inflection point of Gompertz’ curve occurs at a thickness of 37–40% regardless of the substrate and system temperature. A kinetics model proposed by the authors proposes that polymerization occurs in two stages, with the peak growth rate occurring at 37–40% of the ultimate thickness of the film. Maximum growth rates increase with increasing temperature. Higher temperatures, however, result in a slower growth rate for the system. Alternatively, under normal reaction (air, pH 8.5), PDA thickness reaches 50 nm, which takes nearly a few hours up to a few days (Lee et al., 2007). Moreover, the loss of water permeability that is common in other membrane coating procedures can be alleviated by managing the deposition thickness of the PDA layer. This can be achieved through changing PDA deposition timing or the DA concentration in the coating solution, which should be more than 2 mg/ml to produce a PDA film on the surface (Liu et al., 2014). The PDA coating deposition rate can be expedited using ultraviolet irradiation, electrochemical actuation, and oxidant promotion (Jia et al., 2019). A new approach has been proposed by Zhang et al. (Lee et al., 2017), which uses CuSO_4_/H_2_O_2_ to accelerate the PDA deposition rate.

## Evolution of PDA in musculoskeletal regeneration

In recent decades, PDA has attracted significant attention for its unique properties, such as anti-fungal and anti-bacterial properties, adhesion properties, and converting light into heat with high efficiency. This substance has been used in various research fields, such as energy, food industry, and biological applications like cancer treatment, biosensors, drug delivery systems, and TE scaffolds (Xing et al., 2017; Tejido-Rastrilla et al., 2019; Hauser et al., 2020; Shokrollahi et al., 2020). The remainder of this section will discuss the emergence and evolution of PDA in TE and review previous key studies on the applications of PDA in of musculoskeletal TE.

**FIGURE 2:**
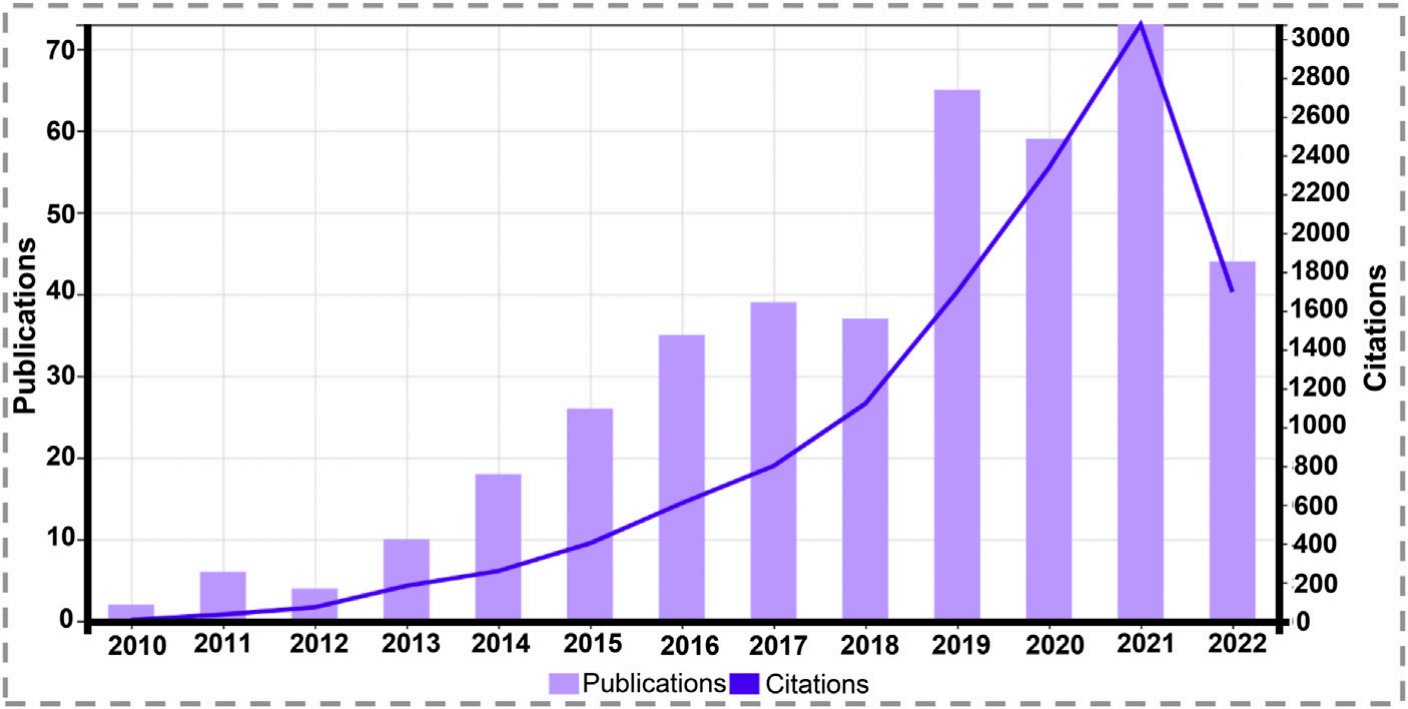
Graph of total scientific papers published and citations per year on “tissue engineering” and “polydopamine coating” since 2010 (source: Web of Science, July 2022).

PDA films with DA polymerization showed robust adhesion capabilities when Lee et al. (Lee et al., 2007) synthesized PDA coatings in 2007. Afterwards, PDA has been used extensively in bioengineering, and the use of this material in the coating of scaffolds for TE has a considerable upward trajectory to date (Figure 2).

Due to the natural adhesive properties of PDA, as well as the possibility of further modifications, PDA can be used to coat bioinert surfaces with potentially increased bioactivity (Barros et al., 2021). By coating the scaffold with PDA, the researchers enhanced cell attachment to the scaffold and brought these adhesive properties into many biomedical applications, especially TE (Lynge et al., 2011; Ghorbani et al., 2022). In developing fields such as organs on a chip and cell sheet engineering, it is also essential to control the spatial distribution of cells on a cell-adhesive patterned structure formed on a non-interacting surface; For this purpose, PDA can be coated in PDMS mold channels to drive cells to adhere to this non-adhere surface (Lynge et al., 2011; Khetani et al., 2020).

Even though bone grafts such as autografts, allografts, and xenografts are commonly used in clinical treatment, they are not ideal for treating bone defects due to their limitations. Ideally, Bone defects should be addressed with a scaffold that facilitates cell proliferation and attachment, exhibits osteodifferentiation capacity, and promotes mineralization (Huang et al., 2016). In bone TE, polymers are an essential material category. Despite the fact that most polymeric biomaterials have suitable properties, they do not provide sufficient osteointegration. The surface characteristics of these polymers can be significantly enhanced by PDA-assisted surface modification. Immobilization of growth factors can substantially improve osteoblast cells’ response to polymeric materials and accelerate the process of material mineralization. As part of bone TE, bone filling materials are commonly used; the PDA coating layer can improve cytoaffinity of the bone cement, resulting in increased fibroblast attachment to the substrate, thereby enhancing cell proliferation (Ko et al., 2013; Cho et al., 2014; Jun et al., 2014; Ghorbani et al., 2019b).

A study conducted *in-vivo* has demonstrated that the PDA coating layer contributes significantly to the biocompatibility of bone substitutes. A poly l-lactic acid (PLLA) film coated with PDA reduces quantum dots’ blood toxicity and enhances their immunogenicity and biocompatibility (Hong et al., 2011). Rim and others modified electrospun PLLA fibers using PDA coating. Several assays were used to determine the effects of PDA modification on hMSC differentiation, including alkaline phosphatase (ALP) activity, collagen matrix formation, osteogenic gene expression, and calcium mineralization. According to SEM images, the PDA-PLLA fibers were coated uniformly with PDA. A comparison of PDA-PLLA fibers with PLLA fibers revealed significant improvements in adhesion, spreading area, ALP activity, and osteogenic differentiation genes. On the basis of these results, the PDA modification in the PLLA was able to control stem cell activity and, thus, serve as a viable carrier for stem cell delivery (Rim et al., 2012).

PDA coating layers can immobilize tender biomolecules that are readily deactivated due to differences in environmental circumstances while exerting no or a small impact on their activity. Through the PDA coating, proteins and growth factors, like bone morphogenic protein-2 (BMP-2), Arg-Gly-Asp peptide, and ALP, can adhere to the surface of scaffold surface. Studies have shown that the PDA layer itself does not influence the activity of these biomolecules other than the molecular weight, incubation time, isoelectric point, and concentration of the biomolecules themselves (Nijhuis et al., 2013). Two studies by Chien and others in 2013 demonstrated that PDA coatings can be used to control cellular behavior. Researchers have found that PDA promotes osteoblast proliferation and calcium deposition and that this effect can be amplified when combined with growth factors (Chien et al., 2013; Chien and Tsai, 2013). In a valuable study, Kao and others (Kao et al., 2015) coated three-dimensional (3D) printed PLA scaffolds with PDA for bone TE. In this study, they directly immersed scaffolds in DA solution to create a PDA coating on them, and this work resulted in increased hydrophilicity and subsequent biocompatibility of scaffolds. PDA-coated samples also revealed decreased bacterial activity and indicate that this easy surface modification induces angiogenesis and osteogenesis differentiation and is a potentially advantageous method to adjust stem cell activity and can act as a helpful carrier for stem cells in bone TE. In a valuable study in 2016, Lee et al. (2016) created bone-tissue regenerative scaffolds by grafting recombinant human BMP-2 (rhBMP-2) to 3D printed polycaprolactone (PCL) scaffolds through coating with PDA. PCL has efficient mechanical properties as a scaffold for bone tissue engineering, and the PDA coating has promoted the hydrophilicity of the PCL and caused efficient immobilization of rhBMP-2, resulting in suitable cell proliferation and osteogenic activity, which suggest that this strategy would be a practical approach for the bone TE field. In the same year, using PDA coating, Wang et al. (2016) immobilized the collagen biomimetic peptide and osteogenic growth peptide on the surface of the poly (lactic-co-glycolic-acid) (PLGA)-hydroxyapatite composite scaffold, which the PDA coating resulted in a notable enhancement in the hydrophilicity of the scaffold and effective peptide immobilization on the substrate, which shows that this causes promotion of MC3T3-E1 cells adhesion, proliferation, and osteodifferentiation compared with the bare substrates and demonstrate that biofunctionalized surfaces with bioactive peptides can supply an exciting method for modification of bone substitutes in musculoskeletal TE.

To achieve sustained release of biomolecules, Yao et al. (2018) proposed that heparin-DA modified graphene foam could be used to enhance BMP-2 release. By coating heparin on the surface of scaffolds with a mimetic DA ligand, the researchers developed 3D graphene foam with robust binding of rhBMP-2. Using this method, durable osteogenic differentiation was achieved as well as the production of an innovative carrier that can accommodate proteins and stem cells. Recently, a valuable study by Ghalandari and others (Ghalandari et al., 2021) indicated the basic mechanism of protein coating formation by coating PDA nanospheres with bovine serum albumin (BSA) and evaluating their binding system and the impact of surface modification on BMSCs’ performance. Their results indicate that the PDA nanospheres coating with BSA resulted in a thin homogeneous appropriate layer for cell adhesion and viability. Furthermore, the expression of osteogenic markers proves a promising ability of the coated PDA spheres for bone tissue engineering.

Since cartilage is an avascular and aneural tissue with restricted chondrocyte proliferation potential, its ability to self-heal is limited. In spite of the fact that many surgical and non-surgical techniques have been developed as treatment or replacement methods for damaged articular cartilage, none of them has been successful to restoring articular cartilage’s native structure and biomechanical properties; therefore, researchers have focused on reconstructing strategies for cartilage TE *in-vitro* (Kessler et al., 2008; Wang et al., 2022).

In 2011 Tsai et al. (2011) demonstrated that PDA-coating on various polymers increased the chondrocytes’ adhesion and proliferation and revealed the potential of this simple strategy for improving cartilage TE scaffolds. In a valuable study in 2013, Cai and others (Cai et al., 2013) coated a 3D printed PCL scaffold with PDA and then grafted it with collagen, making the scaffold hydrophilic and suitable for cell adhesion. Upon these surfaces, chondrocytes are able to attach and maintain a healthy phenotype through the production of cartilage-like extracellular matrix (ECM). PDA was used by Ren et al. (2019) as a coupling agent for grafting chondroitin sulfate onto a PLLA fiber membrane. As a result of their biomimetic surface modifications, the PLLA fibers demonstrate a significant increase in chondrocyte proliferation and attachment, as well as chondrogenic gene expression in rat bone-marrow mesenchymal stem cells (rBMSCs). Additionally, *in vivo* experiments on 6-week-old rabbits demonstrated that the membranes greatly boosted the repair of cartilage defects and the production of hyaline cartilage at the defect zone.

Mechanical properties of the tendon do not reappear following healing, and in most cases, a rupture occurs again. This may happen due to the cells composing the new regenerated tendon are not tenocytes, which are the dominant cells of a healthy tendon (Linderman et al., 2016; Ruiz-Alonso et al., 2021). In this regard, it has been demonstrated that PDA-coated scaffolds can differentiate stem cells into tenocytes. In a study in 2018 (Madhurakkat Perikamana et al., 2018), platelet-derived growth factor (PDGF), a key mediator in tendon repair, was immobilized on a gradient-aligned PDA-coated PLLA nanofiber to regulate the tenogenic differentiation of adipose-derived MSCs (AdMSCs) and found that PDA coating provides a steady display of immobilized PDGF and bioactivity for 2 weeks of incubation which improved the proliferation and tenogenic differentiation of AdMSCs.

The objective of muscle TE is to restore skeletal muscle function that has been impaired by injury, tumor ablations, or congenital defects. Muscle cells’ capability to regenerate under *in-vivo* situations is limited, so muscle TE methods aim to mimic the natural environment *via* scaffolds in conjunction with cells. As a result, several approaches have been developed to enhance the interactions of biomaterials and muscle cells (Madhurakkat Perikamana et al., 2015). For instance, van der Westen and others in 2012 (van der Westen et al., 2012) coated liposomes with PDA and found that this led to higher myoblast viability than unmodified ones. After a year Ku et al. (Ku and Park, 2013) developed PDA-coated aligned PCL nanofibers which is a promising scaffold for skeletal TE, and demonstrated the expression of myogenic proteins and fusion of myoblasts were enhanced compared to their unmodified counterparts.

PDA has been used extensively for in coating neural TE scaffolds in recent years as a result of its desirable properties. Research shows that PDA-modified scaffolds can efficiently absorb neural stem cells, indicating their value for repairing damaged nerves (Yan et al., 2020). In 2012 Yang et al. (2012) demonstrated that PDA modification of substrates promotes the differentiation of stem cells into neurons; in this regard, they immersed various substrates like PLGA and PDMS in a DA solution for 18 h to coat substrates with PDA, and after that, they coated these PDA-coated substrates with adhesion peptides and growth factors such as laminin, fibronectin, and neurotrophic growth factors and then cultured these substrates with neural stem cells. Results demonstrated that PDA is a promising material for effective surface immobilization of proteins and peptides to a manifold variety of biomaterials, and the production of biomimetic scaffolds that promote favorable stem cell activity. On PDA-coated scaffolds, nerve growth factor (NGF) stimulated PC12 cells showed enhanced neuronal differentiation (Bhang et al., 2013). This mussel-inspired bioactive material exhibits exellent potential to modify nerve TE scaffolds, which could be involved in future studies. According to Chen et al. (Chen et al., 2018a), coating a 3D electrically conductive scaffold with PDA increased the hydrophilicity and cytocompatibility of the scaffolds in addition to promoting the proliferation and differentiation of nerve cells, which were enhanced under electrical stimulation. In 2021 Nazeri and co-workers (Nazeri et al., 2021) reported more prolonged growth and adhesion of neurons on PDA-coated PLGA/carbon nanotube scaﬀolds.

## Applications of PDA in musculoskeletal TE

PDA is increasingly being used as a surface modification material, and recently, a number of studies have been conducted on it due to its affordability, availability, and adhesive properties (Ambekar and Kandasubramanian, 2019). This section presents a review of PDA use in coating scaffolds for bioengineering musculoskeletal tissues, with a summary of the findings listed in Table 1.

**TABLE 1:** Summary of PDA coating applications for musculoskeletal tissue engineering.

Tissue type	Scaffold components	Surface modification	Cell source/Animal model	Key properties	Ref
Bone	PCL	PDA	MG-63	Bioactivity	Feng et al. (2021)
				Strong adhesion	
				Cell growth and proliferation	
				Good biomineralization capacity	
				PDA enhanced the hydrophilicity	
	PCL	PDA, BMP-2	MC3T3-E1 osteoblasts	Up to 7 days of profitable BMP-2 retention	Park et al. (2021)
				Enhanced cell growth, proliferation, and osteogenesis	
	PU, GO	PDA	MG-63	Highly increased wettability and water absorption	Ghorbani et al. (2019c)
				Significantly improved proliferation and cell attachment	
	PANI, PVA, PU	PDA	rBMSCs	Enhancement in mechanical properties	Ghorbani et al. (2020b)
				Improved hydrophilicity	
				Great ability in the biomineralization	
				A conductive scaffold with a high PDA facilitates the adhesion and spread of rBMSCs, particularly when electrical stimulation is applied	
	PVDF	PDA	MSCs	Strong adhesion	Xue et al. (2021)
				Exhibiting piezoelectricity in order to modulate the adhesion, spreading, and self-stimulation of cells	
				Improved the hydrophilicity	
	Magnesium	PDA, CDHA, VEGF	MC3T3-E1	Improvement in cell adhesion, proliferation, bioactivity, and angiogenesis	Li et al. (2021)
				Significantly improved the corrosion resistance of Mg	
	PLLA	PDA, Adenosine	hADSCs, Mice	Stimulated osteogenic marker expression and mineralization	Ahmad et al. (2020)
				Displaying osteogenic differentiation in subcutaneous tissue	
				Good biocompatibility	
	PCL	PDA, ALP	L929	Open and aligned pore structure	Ghorbani et al. (2020c)
				High porosity	
				Biocompatibility	
				Modulation of degradation	
	PLGA, *β*-Tricalcium Phosphate	PDA	MC3T3-E1, Mice	*In vitro* test approved that PDA coating improved cell adhesion and osteogenic differentiation	Xu et al. (2019)
				*In vivo* assessments indicate that PDA-coated composite scaffolds perform an acceptable repair effect	
	Cellulose	PDA, Hydroxyapatite	MC3T3-E1	Good distribution of HA	Gao et al. (2022)
				By displaying suitable adhesion and growth characteristics, the scaffold facilitates osteogenic differentiation of the MC3T3-E1 preosteoblast	
	TiO2	PDA, ALP	-	High porosity	Sengottuvelan et al. (2017)
				Suitable compressive strength	
				Deposition of hydroxyapatite	
Cartilage	PCL, PLGA	PDA, Insulin	BMSCs, Rabbits	Increased hydrophilicity, osteogenic differentiation, and proliferation of chondrocytes	Wei et al. (2021)
				Sustained release of insulin after an initial burst release	
				Capability to enhance repair of cartilage and subchondral bone	
	Silk fibroin, hydroxyapatite	PDA, PDGF	Synovial mesenchymal stem cells, Rabbits	Good biocompatibility, proliferation, adhesion, migration, and differentiation of SMSCs into cartilage	Luo et al. (2022)
				Scaffold effectively filled defects and provides an environment for cell proliferation and cell adhesion, migration to the damage site, and differentiates into cartilage	
	PU	PDA	rADSCs, Rabbits	Compared with the natural meniscus, the scaffold had comparable mechanical properties	Ding et al. (2022)
				Cell adhesion, regeneration of meniscus tissue, and osteoarthritis prevention can be enhanced by the modification of scaffolds	
	PLLA, rGO	PDA	ATDC5	The dopamine-modified fibers improved cell adhesion and consequently improved the cell proliferation	Lai et al. (2021)
	HA, Collagen	PDA	BMSCs, Rabbits	Appropriate porous structure	Gan et al. (2022)
				Sufficient mechanical properties	
				Excellent cell affinity	
				Immunomodulatory capabilities	
				Chondrogenic inducibility	
	PLLA, PVA	PDA	-	PDA enhanced the hydrophilicity of surface	Tan et al. (2018)
	Alginate	PDA	L929, CC-H107	Lower degradation rate	Shen et al. (2018)
				Smaller pore size	
				Continuous pore structure	
				Excellent cell attachment	
				Higher compressive modulus	
Muscle	Polypyrrole	PDA	C2C12, Rat	excellent adhesion	Zhou L. et al. (2021)
	Carboxymethyl chitosan			antioxidant abilities	
	F127 poly (citrate-glycol)-polyethylenimine			electrical conductivity	
				Angiogenesis and myofiber formation are enhanced	
				Promoted the regeneration of skeletal muscles *in-vivo* with high effectiveness	
	PLLA	PDA	BMSCs, Mouse	Increased hydrophilicity, cell growth, proliferation, excellent oxidation resistance, and notable *in-vitro* elimination ability of ROS	Xiong et al. (2022)
				*In vivo* approved nanofibers have good histocompatibility	
				Antibacterial ability	
	PCL	PDA, PEDOT:PSS	C2C12	Enhanced myoblasts differentiation and organization	Tang et al. (2020)
				Tunable conductivity and biocompatibility	
	PLLA, Cyclopentanone	PDA	Human MSCs	PDA enhanced hydrophilicity and subsequently biocompatibility	Orlacchio et al. (2022)
				Promote a more efficient transfer of electrical signals among cells during the *in-vitro* regeneration	
Nerve	Cellulose	PDA	NSCs	Promoted NSCs development and adhesion	Yang et al. (2020)
				Spread of NSCs on the electrically conductive scaffold	
	PCL	PDA, dECM	Rat SCs	PDA enhanced the conduits hydrophilicity and improved the amount of dECM coating improving proliferation, cell adhesion, and differentiation	Chen et al. (2018b)
	PCL, Chitosan	PDA, NGF	PC12	Enhanced proliferation and cell attachment in a certain direction	Afrash et al. (2021)
				Enhanced the hydrophilicity of nanofibers	
	PCL/CNTs	PDA, BDNF	Rat SCs, Sprague-Dawley rat	Increased the expression of genes related to myelination and proliferation in SCs	Pi et al. (2022)
				Sustained release BDNF	
	Chi	PDA, Exosomes	BMSCs, SCs, Sprague-Dawley rat	Efficient adhesion for exosomes sustained exosome-release	Li et al. (2022a)
				Better proliferation of SCs	
	Chi	PDA	RSC 96 cells, Rats	Sufficient biocompatibility	Yang et al. (2022)
				Perfect biodegradability	
				Scaffolds distinctly facilitated the sciatic nerve regeneration	
				Remarkably decreased the inflammatory response	
				Scaffolds inhibited painful neuroma formation	
	Chi	PDA, BDNF and VEGF mimetic peptides	SCs, HUVECs, Sprague-Dawley rat	Encourage the proliferation of endothelial cells and SCs sustained release of the mimetic peptides	Li et al. (2022b)
				conduits had enhanced axonal repair, remyelination, and neuronal recovery *in-vivo*	
Tendon	PDS II, Ethilon	PLGA, PVA, PDA, bFGF, VEGFA	Tendon primary cells, Chicken, Sprague-Dawley rat	Sustained-release of growth factors	Zhou Y. L. et al. (2021)
				Promote tendon healing	
	PDS II, Ethilon	PLGA, PEI, PDA, bFGF, VEGFA	Primary Tenocytes, Chicken, Rat	Increasing gene delivery and growth factor expression	Zhou et al. (2019)
				Promote tendon healing	
	Silk fibroin	PDA, KGN	BMSCs, Sprague-Dawley rat	Antioxidant functionality	Chen et al. (2021)
				Reduction of oxidative stress and inflammation	
				Induced interface tissue remodeling	
				Promotes formation of fibrocartilage	
				Integration of the tendon with the bone in an effective manner	
	PET	PDA, Nano-Hydroxyapatite, Silver	NIH3T3, MG-63	Enhanced cell proliferation	Wu et al. (2021)
				Significant antibacterial properties	

### Bone regeneration

**FIGURE 3:**
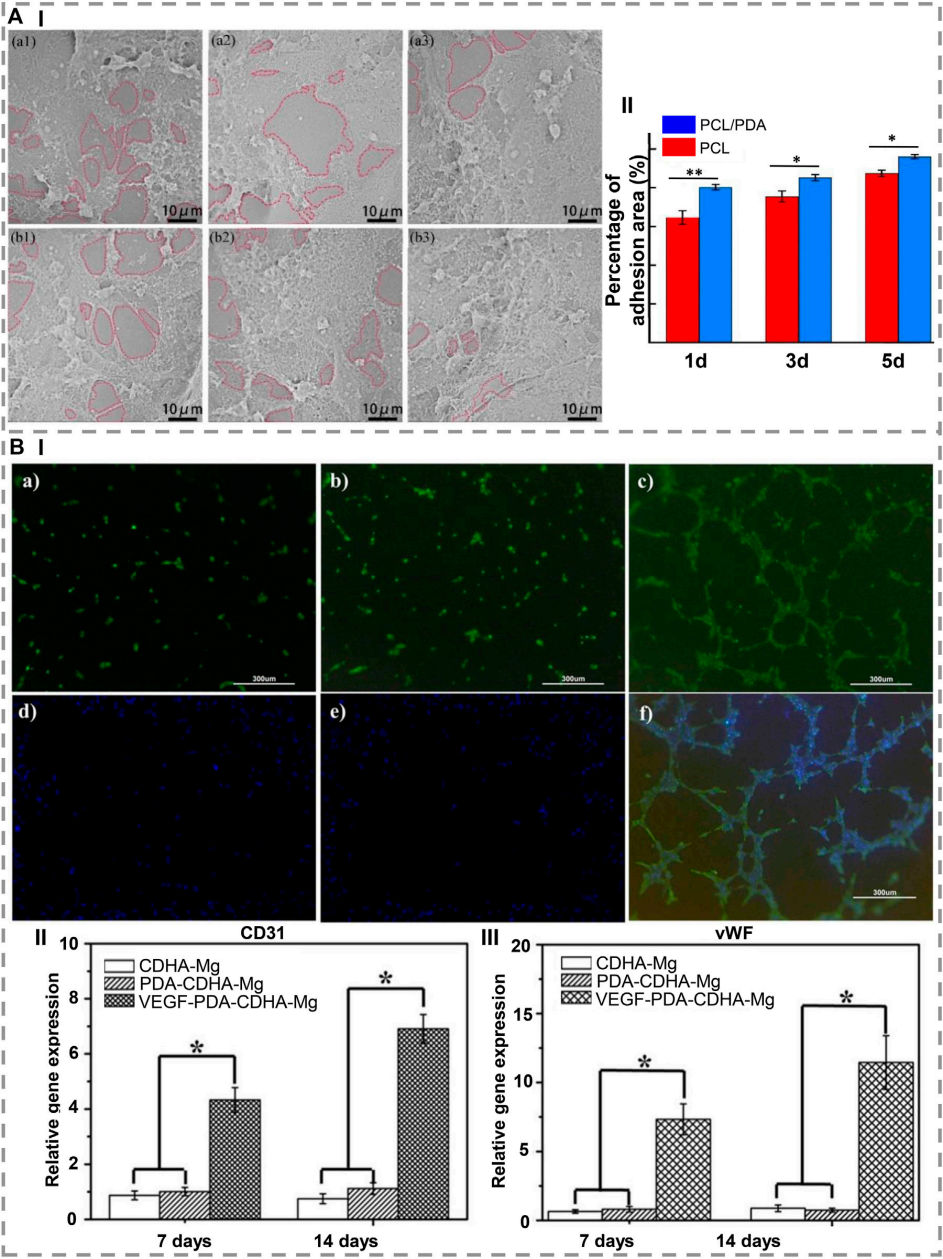
**(A) (I)** SEM images show that, after 1, 3, and 5 days of culture, (a1-a3) PCL and (b1-b3) PCL/PDA scaffolds are capable of adhesion and proliferating cells. (II) After 1, 3, and 5 days of cultivation, the adhesion area of the PCL scaffold and the PCL/PDA scaffold is shown. ∗ indicates a statistical difference (at the same time, *p < 0.05, **p < 0.01) [Reproduced under the terms of the Creative Commons Attribution-NonCommercial-NoDerivatives 4.0 International (CC BY-NC-ND 4.0) license. (Feng et al., 2021) Copyright 2021, Elsevier]. **(B) (I)** In vitro development of microtubule in rbMSCs cultured on **(a)** CDHa-Mg, **(b)** PDA-CDHa-Mg, and **(c)** VEGF-PDA-CDHa-Mg after 2 weeks. **(d–f)** Expression of alpha-smag by immunofluorescence in rBMSCs grown on CDHa-Mg, PDA-CDHa-Mg, and VEGF-PDA-CDHa-Mg after 2 weeks. For 7 and 14 days, rBMSCs were cultured on CDHa-Mg, PDA-CDHa-Mg, and VEGF-PDA-CDHa-Mg to determine the expression levels of (II) CD31 and (III) vWF. (n ​= ​3, ∗ indicate statistical significance, p ​< ​0.05) [Reproduced under the terms of the Creative Commons Attribution-NonCommercial-NoDerivatives 4.0 International (CC BY-NC-ND 4.0) license. (Li et al., 2021) Copyright 2021, Elsevier].

The lack of bioactivity of artificial implants prevents them from osseointegrating well with host bone tissue. Due to this restriction, it cannot be used to regenerate bone. Feng et al. (2021) modified PCL particles with PDA, fabricated them on a scaffold using selective laser sintering, and demonstrated that PDA imposed a negative charge on the scaffold surface, which attracted Ca^2+^ ions, forming an apatite layer. Also, the hydrophilic groups of PDA reduced water contact angles to an extent, leading to rapid cell proliferation and adhesion as well as protein absorption. Therefore, it is beneficial for bone TE applications (Figure 3A). Similarly, Park et al. (2021) coated the 3D scaffold with PDA and then deposited hydroxyapatite nanoparticles *via* biomimetic mineralization to immobilize BMP-2. Using PDA coatings and hydroxyapatite layers containing BMP-2, *in-vitro* results were positive for osteoblast proliferation and indicated an excellent potential for bone repair.

Ghorbani et al. (2019c) study investigated electrospun polyurethane (PU)-graphene oxide (GO) scaffolds covered with PDA. Despite the fact that GO incorporated into electrospun scaffolds improved physical and biological properties, PU-GO-PDA scaffolds demonstrated significantly enhanced features, including wettability (contact angle: 54.53° ± 1.34°), water absorption (14% increase in 24 h), MG63 cell attachment, and proliferation. When compared with the control group and the PU-GO group, the PU-GO-PDA scaffold significantly increased proliferation. In addition, the PU-GO-PDA scaffold significantly increased ALP expression, about 70 U/L compared to the control group, about 45 U/L in 7 days, indicating osteogenic differentiation. The PU-GO-PDA scaffold appears to be an effective substrate for bone regeneration *in-vivo*. The following year, Ghorbani et al. (2020b) studied the physicochemical, mechanical, and *in-vitro* characteristics of modified electrospun PU-polyaniline (PANI) scaffolds functionalized with PDA as well as the influence of substrate composition chemistry and hydrophilicity on a dense and homogeneous functionalization. In this approach a uniform, dense, and homogeneous coating of PDA applied to the surface of the PU-PANI/PVA scaffold in order to improve its mechanical properties, hydrophilicity, absorption capacity, and mass-loss rate. It was found that the PDA-modified PU-PANI/PVA constructs were able to biomineralize hydroxyapatite-like layers, which is critical for bone regeneration. On high PDA substrates, rBMSCs were shown to adhered and spread well, particularly when electrical stimulation was applied. ALP was expressed, and collagen I was secreted at a suitable level after the membrane was coated with PDA and electrically stimulated.

In order to investigate the effects of polymerizing DA on cell adhesion and electromechanical signaling in poly vinylidene fluoride (PVDF) membranes, Xue et al. (2021) modified PVDF membranes as a bone healing material by polymerizing DA. The results demonstrated that seeded MSCs adhered and spread better on polarized PVDF membranes. The PDA surface modification also improved the hydrophilicity and induced cell focal adhesion formation, thus increasing piezoelectric self-stimulation and intracellular calcium transients. Regarding bone regeneration, Li et al. (2021) immobilized vascular endothelial growth factor (VEGF) on Mg, and as a result, a functional VEGF-PDA-CDHa (Ca-deficient hydroxyapatite) composite coating was successfully applied to Mg. As an intermediate layer between Mg and VEGF, the PDA coating serves as a receptor site for VEGF. This facilitates the development of angiogenesis during the early period of implantation. The VEGF-PDA-CDHa coating on MC3T3-E1 cells on Mg demonstrated great biocompatibility as well as remarkably increased cell adhesion and proliferation (Figure 3B).

According to Ahmad et al. (2020), PDA can be used as a one-step coating to protect adenosine on biomaterial surfaces. In order to create human-adipose-derived stem cell (hADSC) spheroids for bone regeneration, the researchers introduced an adenosine-ligand onto engineered fibers that were inspired by the ECM, using PDA chemistry. Using an adenosine-ligand-engineered fiber, the adenosine 2 b receptor (A2bR) was stimulated, osteogenic differentiation was stimulated, adipogenic differentiation was inhibited, and then engineered spheroids were injected into mice to induce osteogenic differentiation and to regenerate bone in calvarial defects.

In another exciting study, Ghorbani and others (Ghorbani et al., 2020c) fabricated a PCL scaffold through a unidirectional freeze-casting method and modified its surface with PDA, which resulted in increased hydrophilicity and water uptake leading to the modulation of the timing of the PCL scaffold degradation. Also, *in-vitro* tests revealed that the PDA-modified PCL scaffold appeared to display good biocompatibility and cell proliferation, with more than 85% of L-929 viable cells, and could be a promising platform for further studies in bone tissue engineering.

3D-printed scaffolds of PLGA/*β*-tricalcium phosphate were coated with PDA by Xu et al. (2019). As a result of the coatings, the mechanical parameters and pores-related features of the multilayer scaffolds are not altered, and the hydrophilicity of the surface is clearly enhanced. In addition to promoting the proliferation and adhesion of cells, the modified surface also promotes osteogenesis through the increase in PDA concentrations. As a result of *in-vivo* experiments, scaffolds containing a greater amount of PDA coating demonstrate a more remarkable ability to stimulate bone formation.

Deacetylated porous cellulose acetate microspheres (DPCA) with a 3D porous structure are used by Gao et al. (2022) to synthesize hydroxyapatite suspended within a PDA coating. There is a unique porous structure of the DPCA-PDA-hydroxyapatite microspheres (DPPH) scaffold, as well as a uniform coating of PDA and a well-distributed hydroxyapatite crystal distribution throughout the scaffold. MC3T3-E1 preosteoblasts adhered to and grew abundantly on calcium compounds, enhancing osteogenesis in the one-step modification process. With the aid of a facile preparation method, DPPH was fabricated, demonstrating its potential as a scaffold for supporting bone regeneration.

Titanium dioxide (TiO_2_)-based scaffolds have a great mechanical property besides their biocompatibility for bone tissue engineering. Still, their bioactivity is not enough, and in this regard, Sengottuvelan and coworkers (Sengottuvelan et al., 2017) fabricated TiO_2_ scaffolds by foam replica molding and then coated them with ALP using PDA to enhance their bioactivity, which resulted in a high porous scaffold with about 97% porosity and compressive strength of ∼2.7 MPa. ALP immobilizing on the surface was resulting in the deposition of HA and demonstrated that this method could enhance the bioactivity of TiO_2_ scaffolds.

### Cartilage regeneration

Cartilage and subchondral bone have a low ability to regenerate and repair; for this reason, researchers such as Wei et al. Wei et al. (2021) coated 3D printed PCL scaffolds with PDA, which increased PCL hydrophilicity, biofunctionality, and biocompatibility as well as improving cell adhesion to the scaffolds. Moreover, it helped scaffold adsorb PLGA nanoparticles loaded with insulin. They discovered that the insulin-PLGA/PDA/PCL scaffolds showed better healing, more stability, and improved cartilage repair *in-vivo* when used on cartilage and bone. In another study, Luo et al. (2022) constructed a three-layer osteochondral bionic scaffold using hydroxyapatite and silk fibroin. Further, they used PDA on the scaffold, increasing the release and loading of the platelet-driven growth factor for a considerably more extended time than the other group resulting in the differentiation of synovial MSCs into cartilage. They showed that *in-vivo* experiments, the PDA-modified scaffolds were significantly better at enhancing adhesion and proliferation of cells, leading to the better repair of osteochondral defects and cartilage regeneration. Ding et al. (2022) coated the PU 3D printed scaffold with PDA to enhance biocompatibility and hydrophilicity as well as increase the ability to recruit endogenous MSCs. In addition to reducing the water contact angle of scaffolds, PDA-modification resulted in higher cell viability, uniform adhesion, and improved cell proliferation. Furthermore, implanted scaffolds demonstrated acceptable gene expression for chondrogenic differentiation and cartilage regeneration. In a parallel study, Lai et al. (2021) increased the hydrophilicity of PLLA/graphene electrospun scaffolds with a layer of PDA coating which directly increased cell adhesion, proliferation, and chondrogenic differentiation.

**FIGURE 4:**
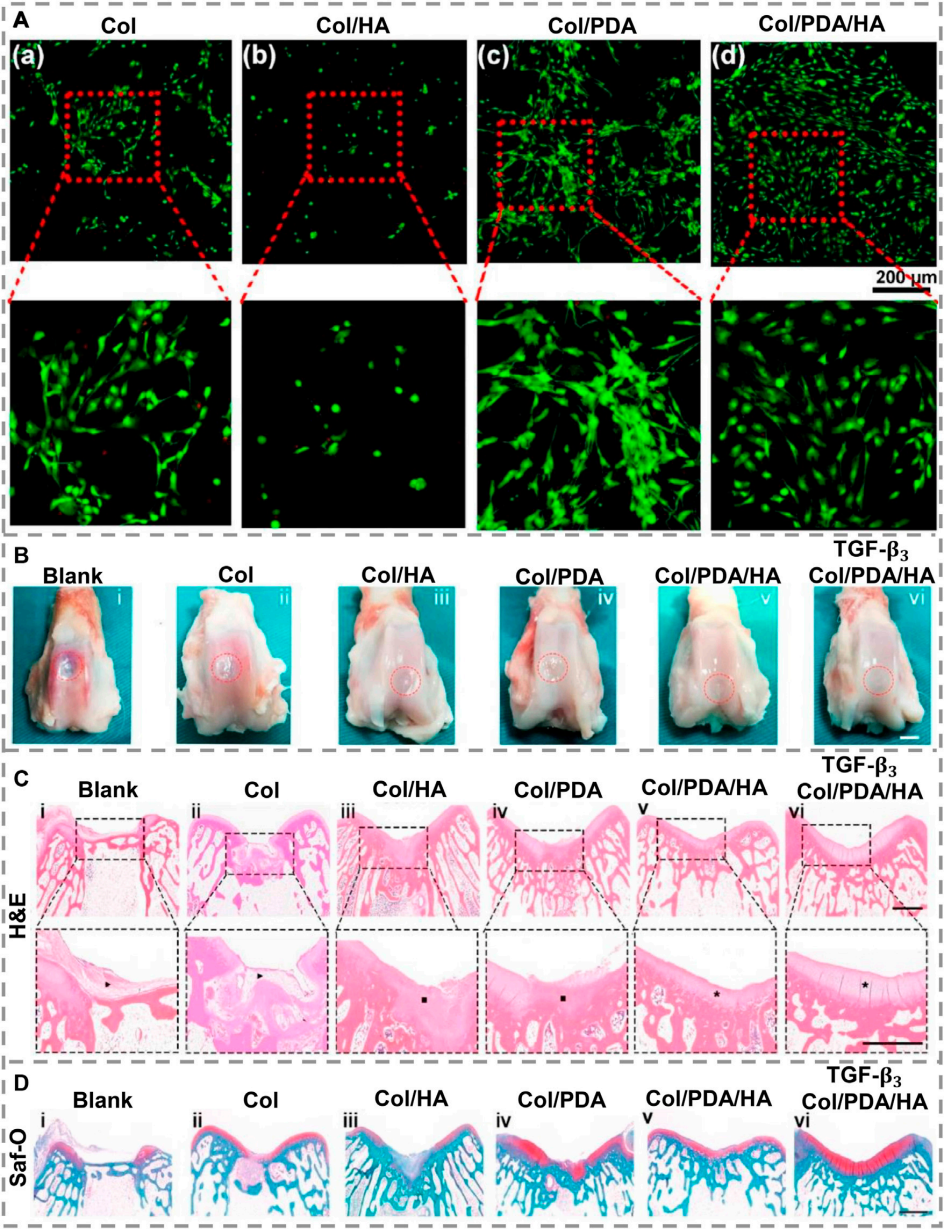
**(A)**. In-vitro cytocompatibility assessment of scaffolds after 3 days of cultivation in primary medium **(a–d)**. A green stain was applied to live cells and a red stain to dead cells. **(B)**. Hydrogel scaffolds for cartilage repair in-vivo. After 12 weeks of post-healing, a macroscopic examination of regenerated cartilages was conducted on different groups. **(C)**. H&E and **(D)**. Staining of post-sacrifice defects with Safranin-O. The dashed lines indicate the approximate location of defect sites. Fibrous tissue is indicated by black arrows, while cartilage tissue is indicated by black squares. A black asterisk indicates well-organized cartilage. [Reproduced under the terms of the Creative Commons Attribution-NonCommercial-NoDerivatives 4.0 International (CC BY-NC-ND 4.0) license. (Gan et al., 2022) Copyright 2022, Elsevier].

To mimic the ECM of cartilage, a PDA-modified hyaluronic acid (PDA/HA) was integrated into a collagen substrate to achieve a collagen/PDA/HA scaffold (Gan et al., 2022). This scaffold provides cell attachment with 3D framework support by dually cross-linking the collagen substrate to create matrices with durable mechanical characteristics and resembling the ECM’s connective fibers. By functionalizing HA using PDA, adhesive catechol groups are grafted to HA chains, which mimic the ECM’s component glycosaminoglycans, increasing cartilage-specific gene expression and enhancing cell adhesion, which can efficiently direct BMSCs to osteogenic differentiation. The collagen/PDA/HA hydrogel scaffolds displayed remarkable cell affinity and cytocompatibility. To illustrate this, BMSCs were cultured on the scaffold, and, for comparison, it also individually cultured on scaffolds made of hydrogel components. Figures 4Aa,b show that only a small number of cells were present on scaffolds that do not contain PDA. On the other hand, there are considerable numbers of cells on the surface of scaffolds containing PDA. The enlarged images revealed that most cells on the PDA-containing scaffolds showed usual spindle-like morphology (Figures 4Ac,d), confirming that the PDA segment in the hydrogels promoted cell spreading as well as adhesion and demonstrating that PDA functionalization enhanced cell affinity for HA. To assess its capability to support cartilage regeneration *in-vivo*, collagen/PDA/HA scaffolds were implanted into rabbit cartilage defects. As positive groups, transforming growth factor-β3 (TGF-β3)-loaded hydrogels were implanted too. Macroscopic gross morphology analysis revealed that regenerated tissues mostly filled the defects in all groups 12 weeks after implantation (Figure 4B). Collagen/PDA/HA treated group filled defects by forming cartilage-like tissue maintaining a uniform and homogeneous surface that merged well with host cartilage (Figure 4Bv), which was similar to the TGF-β3-loaded group (Figure 4Bvi). The quality of restored cartilage was assessed by histological analysis after H&E (Figure 4C), safranin-O staining (Figure 4D) and immunohistochemistry (IHC) for collagen type II and aggrecan. In the collagen/PDA/HA group, the injury was filled with a uniform and dense cartilage layer congruent with the neighboring host cartilage (Figure 4Cv). In comparison with normal cartilage, the collagen/PDA/HA hydrogel scaffold showed comparable Safranin-O staining. It was observed in IHC that both collagen II and aggrecan were severely stained in the collagen/PDA/HA groups, as well as in the TGF-β3-loaded groups, indicating that collagen/PDA/HA hydrogel groups produced an adequate amount of ECM and integrated cartilage repair. Furthermore, these results demonstrate that new cartilage formed by collagen/PDA/HA was phenotypically stable. *In-vivo* investigations indicated that collagen/PDA/HA hydrogel scaffolds were more capable of repairing cartilage tissue than collagen/HA scaffolds, resulting in higher quality cartilage tissue than collagen/HA scaffolds.

During the fabrication of their scaffold, Tan et al. (2018) used PDA as a surface modification material and suggested it for cartilage TE. In this investigation, the surface of porous PLLA membrane was coated with PDA, which resulted in a water contact angle of 58.51° ± 3.50°, indicating that PDA led to a more hydrophilic surface. It was observed that porous PLLA membranes without a coating of PDA formed water droplets on their surfaces. The PDA coating instantly absorbed the water droplet into the membrane architecture, demonstrating the benefits of this coating. In an impressive study, Shen and others (Shen et al., 2018) utilized DA and PDA to create a new alginate scaffold *via* chemical and physical interactions to fabricate a scaffold with optimal mechanical features and biological activity for cartilage TE. To fabricate an alginate-DA/PDA scaffold, alginate has been modified with DA through esterification and then knotted with PDA nanoparticles. In addition to its excellent biocompatibility and cell attachment, this scaffold showed promise for cartilage TE since it exhibits continuous pore structure, reduced pore size, better compressive modulus, and a lower degradation rate than the one of other scaffolds.

### Muscle regeneration

**FIGURE 5:**
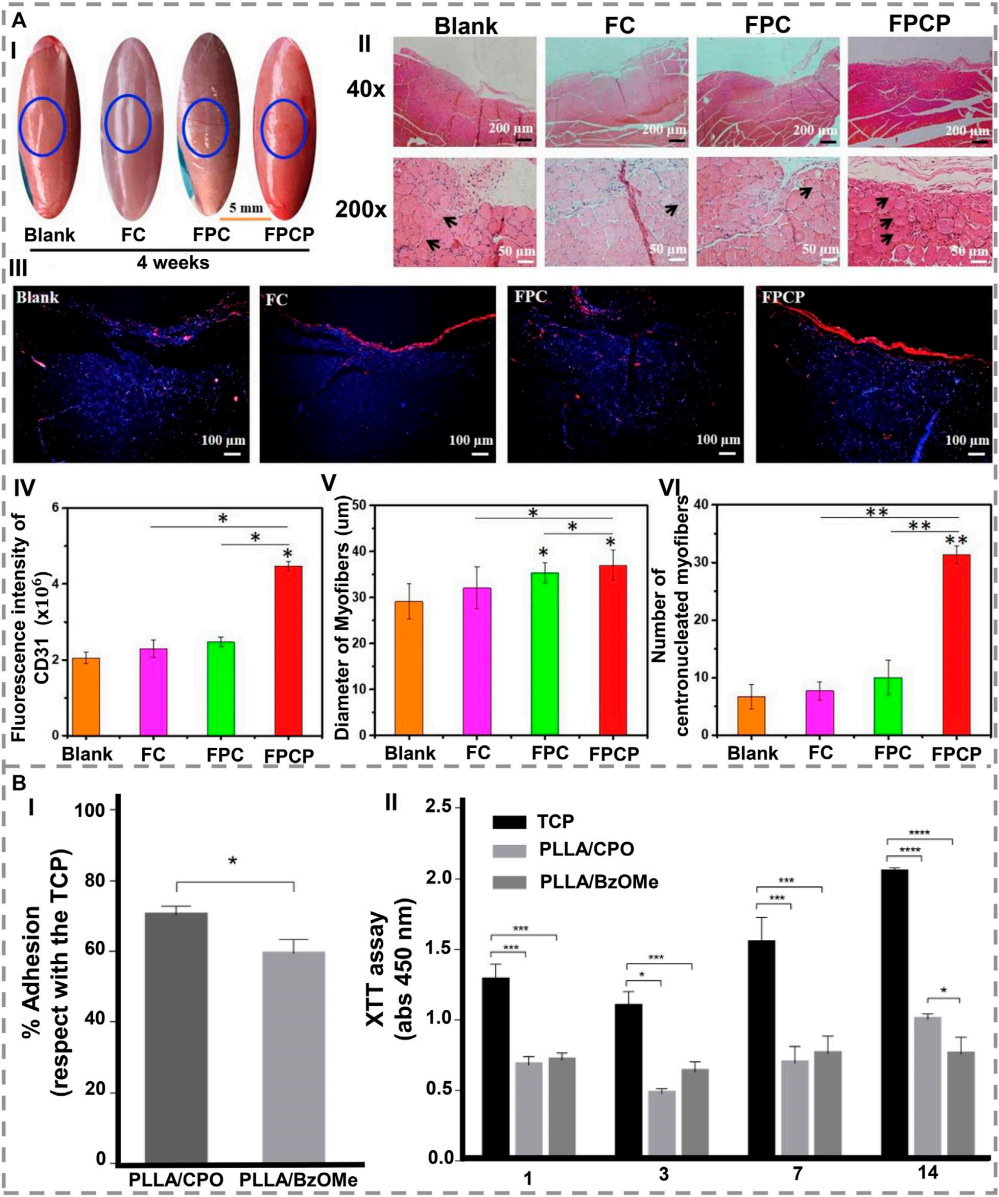
**(A)** A period of 4 weeks of treatment with FPCP hydrogel led to the regeneration of skeletal muscle in-vivo (blue: a sham operation). **(I)** The following images describe the skeletal muscle defect in detail. (II) Images of the defect area stained with H&E and showing centronucleated myofibers. (III) CD31 stained images (blue represents nuclei, red represents CD31). (IV) Quantitative analysis of CD31. (V) Measurements of myofiber diameter, and (VI) numbers of regenerated myofibers (centronucleated myofibers) near the defect site. By using ImageJ software, three randomly selected fields of each section and three sections per specimen were analyzed (*p < 0.05 and **p < 0.01) [1. Reproduced under the terms of the Creative Commons Attribution-NonCommercial-NoDerivatives 4.0 International (CC BY-NC-ND 4.0) license. (Zhou L. et al., 2021) Copyright 2021, Elsevier]. **(B)**. In-vitro evaluation of PLLA/CPO and PLLA/BzOMe aerogels. **(I)** Cell adhesion after 24 h. Results illustrate the percentage of adhesion concerning the control (TCP). (II) Cell proliferation after 1, 3, 7, and 14 days (*p < 0.05; ***p < 0.01; ****p < 0.0001) [2. Reproduced under the terms of the Creative Commons Attribution 4.0 International (CC BY 4.0) license. (Orlacchio et al., 2022) Copyright 2022, MDPI].

Skeletal muscle regeneration still faces the challenge of scattered injury and diseases. Zhou L. et al. (2021) developed polypyrrole (PPy) covered by PDA in a crosslinked nanocomposite hydrogel that displays a thermo-responsive gelation property. Their hydrogel exhibited excellent adhesion, antioxidant abilities, and electrical conductivity for *in-vitro* and *in-vivo* stimulation of myoblast differentiation and repair of skeletal muscle (Figure 5A). Xiong et al. (2022) have reported the synthesis of PDA and PLLA nanofibers. Following *post-in situ* polymerization, PDA can be uniformly coated on PLA nanofiber, and these composites exhibit superior mechanical properties, hydrophilic properties, enhanced oxidation resistance, and near-infrared photothermal properties. PDA/PLLA scavenges reactive oxygen species (ROS) and promotes tissue repair *in-vitro* as well as good histocompatibility and increase of fibroblasts number after 7 days implantation of PDA/PLLA into mouse back muscles.

The surface properties of the matrix influence myogenic differentiation and maturation. Hence, DA self-polymerization can modify nanofiber matrices before coating them with conductive polymers and promote myoblast differentiation by upregulating myogenic markers. Therefore, Tang et al. (2020) fabricated a new stimulus-responsive conductive nanocomposite structure in which they first coated PCL fibers with PDA and then with poly (3,4-ethylene dioxythiophene): poly (styrene sulfonate). The results showed that the DA coating successfully increased hydrophilicity while minimally affecting fiber morphology. Based on the presented results, it has been demonstrated that aligned conductive fibrous matrices can be used to restore skeletal muscle. In a recent study, Orlacchio and others (Orlacchio et al., 2022) observed that PDA enhanced the hydrophilicity of PLLA aerogels and improved their bioactivity, indicating that this could allow electrical signals to be transferred amongst the cells resulting in an effective scaffold for tissues with bioelectrical functionalities such as muscle (Figure 5B).

### Nerve regeneration

**FIGURE 6:**
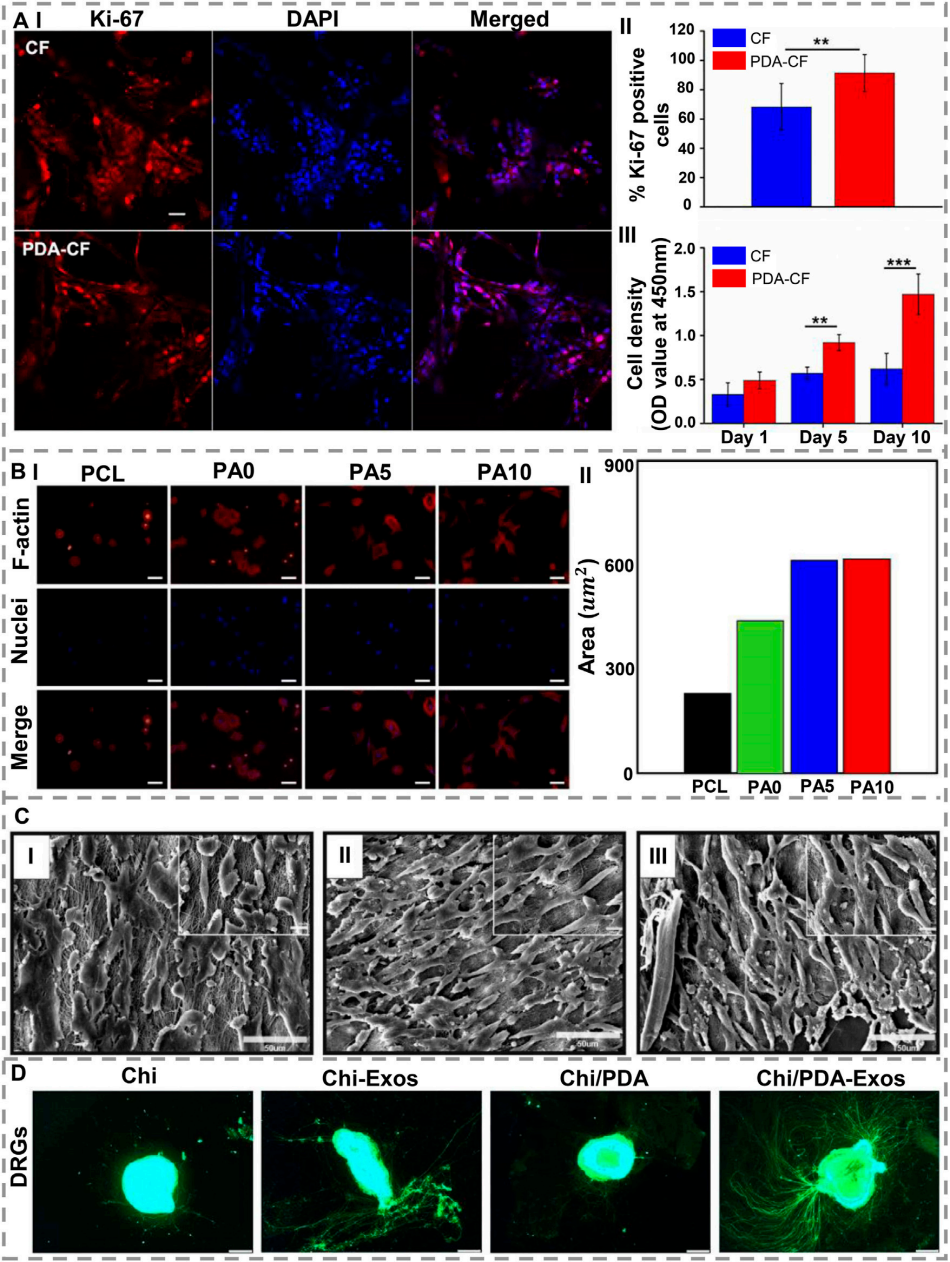
**(A) (I)** Confocal laser scanning microscopy with immunostained NSCs (Ki-67 and DAPI). (II) During the fifth day of culture, Ki-67-positive cells observed. (III) CCK-8 relative viability assay. Scale bar = 50 μm (**p < 0.005; ***p < 0.0005) (reproduced content is open access) [1. Reproduced under the terms of the Creative Commons Attribution 4.0 International (CC BY 4.0) license. (Yang et al., 2020) Copyright 2020, Frontiers]. **(B) (I)** Staining of nuclei (blue) and F-actin (red), and (II) the site quantification following 3 h seeding of RSCs on dECM/PDA-coated PCL scaffolds. Scale bar= 200 µm [2. Reproduced under the terms of the Creative Attribution 4.0 International (CC BY 4.0) license. (Chen et al., 2018b) Copyright 2018, MDPI]. **(C)**. SEM images of PC12 cells on **(I)** PCL-Chitosan (Aligned) nanofibers; (II) PCL-Chitosan-PDA nanofibers; (III) PCL-Chitosan-PDA-NGF nanofibers. Nanofibers immobilized with NGF allowed cells to spread and grow longer [3. Reproduced under the terms of the Creative Commons Attribution 4.0 International (CC BY 4.0) license. (Afrash et al., 2021) Copyright 2021, AMG]. **(D)**. After 7 days of treatment with Chi/PDA-Exos, neurite growth is enhanced in DRGs. As a result of the application of BMSC-Exos, DRG axons (green, Alexa Fluor 488) were measured with different conduits and their length was enhanced. In comparison with Chi alone, treatment with Chi/PDA-Exos significantly increased the length of the DRG axons. Scale bars = 50 μm [4. Reproduced under the terms of the Creative Commons Attribution-NonCommercial-ShareAlike (CC BY-NC-SA) license. (Li et al., 2022a) Copyright 2022, Wolters Kluwer Medknow].

The main issue concerning nerve repair is the lack of transplantable nerve tissue, and as a widely used material in surface modification, PDA has been utilized to modify nerve conduits to repair and regenerate injured nerves. For this reason, Yang et al. (2020) fabricated a PDA-functionalized carbon microfibrous electrospun scaffold, and they found that the expression of Ki-67 protein has been expanded by PDA, which consequently increased the cell adhesion and proliferation of neural stem cells. Additionally, they realized that neural stem cells (NSCs) formed more rapidly on the PDA-carbon microfibrous surface than on the carbon microfibrous scaffold (Figure 6A). According to other research, Chen et al. (2018b) deposited PDA directly onto 3D printed scaffolds constructed from PCL. *In-vitro* experiments demonstrated that the existence of PDA improved the hydrophilicity of nerve conduits, and increased Schwann cells (SCs) growth, adhesion, differentiation, proliferation, and cell functions, as well as mechanical properties (Figure 6B). Afrash et al. (2021) fabricated PCL/chitosan nanofibers with PDA and found that coated DA layer conjugated NGF onto the scaffold, improved cell adhesion and proliferation by immobilizing NGF (Figure 6C). Recently, Pi et al. (2022) modified PCL/carbon nanotube fibrous scaffolds with PDA in order to obtain scaffolds that release sustained levels of brain-derived neurotrophic factor (BDNF). These scaffolds were used to culture SCs *in-vitro*, and they enhanced peripheral nerve regeneration, stimulating cell proliferation and myelination-related gene expression. Furthermore, these scaffolds demonstrated similar regenerative properties of peripheral nerves *in-vivo* as autologous nerve grafting on a rat sciatic nerve defect model of a 10-mm length. (Li et al. (2022a) developed a sustained, stable release of MSC exosomes using PDA-modified chitin (Chi) conduits. The exosomes isolated from rat MSCs stimulated SCs to proliferate and reprogrammed them toward a repair phenotype *in-vitro*. A PDA-modified Chi conduit containing MSC-derived exosomes was found to augment neurite growth in the dorsal root ganglia of rats with sciatic nerve defects (Figure 6D). The anti-inflammatory activity of PDA was also illustrated in a study conducted by Yang et al. (2022) that modified a Chi-based nerve guidance conduit and showed its ability to inhibit neuromas during nerve regeneration. In addition, PDA promoted cell viability and protuberance expansion. Based on *in-vivo* trials, this scaffold facilitated sciatic nerve regeneration with complete biodegradability and displayed an identical amount of muscle fibers and myelin thickness compared to autografts.

VEGF and BDNF affect peripheral nerve regeneration by providing a supportive microenvironment (Lopes et al., 2017; Xia and Lv, 2018). The clinical benefit of these factors is limited due to rapid degradation *in-vivo* and obstructing axonal growth when used at supraphysiological doses. There has been recent development of bioactive peptides that mimic neurotrophic factors and can stimulate the equivalent receptors but have modified sequences and forms, making them a valuable substitute for the original growth factors (Lu et al., 2018; Rao et al., 2020). Li et al. (2022b) developed PDA-modified Chi conduits loaded with BDNF mimetic peptides and VEGF mimetic peptides and showed that adding PDA to this conduit caused higher biocompatibility and sustained release of peptides. In rat models of sciatic nerve injury, these conduits bridged a 2-mm gap among the nerve stumps. As a result of applying Chi/PDA-Ps conduits, rats were found to have improved motor function and less atrophy in their gastrocnemius muscles. A further restoration of nerve conduction function and remyelination was demonstrated by electrophysiological and microstructural results.

### Tendon regeneration

**FIGURE 7:**
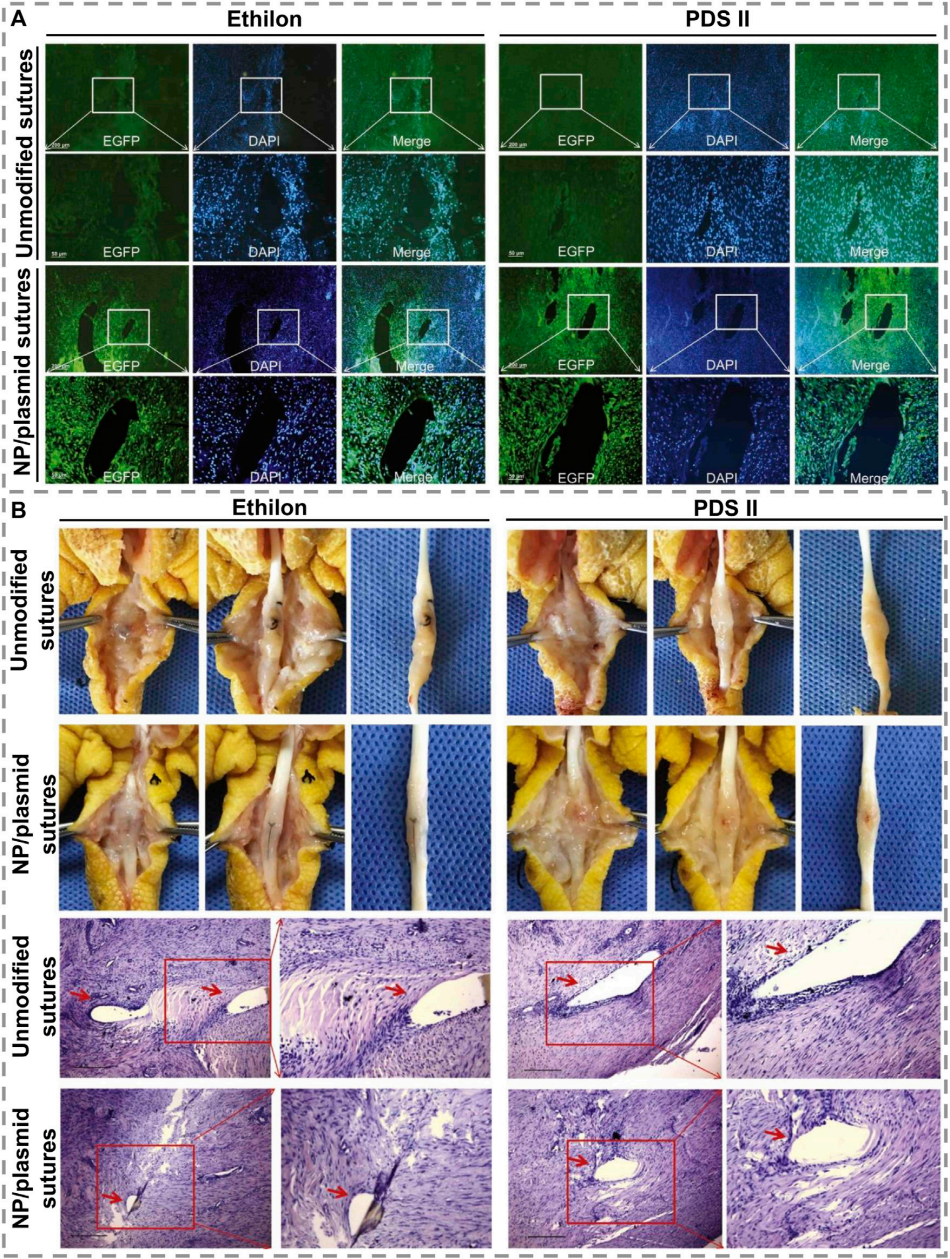
**(A)**. Displaying EGFP expression images of modified and unmodified two kinds of sutures, Ethilon and PDS II, in the tendon tissues 1 week after surgery. Scale bars = 50 μm. **(B)**. Images of adhesion morphology in the unmodified or modified sutures group 6 weeks after treatment and histology of tendons treated with modified sutures. Scale bars = 100 μm [Reproduced under the terms of the Creative Commons Attribution-NonCommercial-NoDerivatives 4.0 International (CC BY-NC-ND 4.0) license. (Zhou et al., 2019) Copyright 2019, Elsevier].

Tendons have a unique composition of their ECM, in addition to being hypocellular and hypovascular, making them less capable of healing. Zhou Y. L. et al. (2021) found that growth factor-loaded PLGA nanoparticles adhered uniformly to PDA-modified suture surfaces and accelerated tendon repair. Even after being sutured into the tendon, the nanoparticles remained on the sutures, allowing proteins to penetrate the tendon tissues. Both chicken and rat tendon injury models have been studied in which basic fibroblast growth factor (bFGF)-releasing and VEGF-releasing sutures resulted in significantly higher tendon strength than those repaired with control sutures. In another interesting research on sutures for tendon regeneration, Zhou et al. (2019) coated nanoparticle/plasmid complex on PDA-modified sutures to transfer the growth factor genes into injured tendon tissues to promote healing. PDA-modified sutures can uniformly and tightly absorb nanoparticle/plasmid complexes and deliver and transfer them into tendon tissue to effectively transfect genes into tendon cells, significantly increasing the expression of growth factors in tendon tissues. As it showed in Figure 7A, the nanoparticle/plasmid complexes can detach from the modified sutures and spread out in tendon tissues after stitching and enhanced EGFP expression, while no EGFP-positive cells were observed in the unmodified sutures group. Figure 7B displays that *in vivo* experiments and histology of repaired tendons indicate that all of the repaired tendons had no significant inflammatory reactions or necrosis 6 weeks after surgery and indicate that these modified sutures are a promising tool for the treatment of injured tendons. As a result of modifying PDA and kartogenin carboxyl groups, Chen et al. (2021) prepared multi-functionalized nanofibrous scaffolds with amide bonds on the silk fibroin nanofibers’ surface. When used in the early stages of tissue repair, multifunctional nanofibers reduce oxidative stress and inflammation as well as inducing remodeling and regeneration. As a result of the ECM’s biological properties, the nanofiber scaffold also facilitated cell differentiation and tissue formation. Nanofibers modified with PDA and KGN enhanced fibrocartilage regeneration and improved integration of tendons and bones *in-vivo*, leading to good biomechanical function. Wu et al. (2021) used PDA nano-layers, silver, and nano-hydroxyapatites to eliminate antibiotics after surgery by binding to polyethylene terephthalate (PET) ligament’s biological inertness. As DA undergoes only a mild polymerization reaction, PET remains chemically and mechanically stable. NIH3T3 cell culture for fibroblast growth and *staphylococcus aureus* was used to test PET artificial ligament’s biocompatibility and antimicrobial effects.

## Perspective summary

PDA has excellent biocompatibility, hydrophilicity, and adhesion properties. Therefore, this technology has been widely used in a variety of fields and is expected to be further applied in TE and regenerative medicine. PDA can easily be applied to different biomaterials and enhance their biological and mechanical properties using the polymerization process. Over the past few years, PDA has been successfully applied in musculoskeletal TE. It has been demonstrated that surface modification of scaffolds by PDA is an effective method for controlling the behavior of cells, which is based on the super-hydrophilic properties imparted to the scaffold surface which serves as a cell carrier for musculoskeletal TE.

Despite extensive research on PDA, only a few investigations have studied the impacts of PDA on musculoskeletal tissue engineering and its structure and polymerization process remain little understood and should be further investigated. It would also be beneficial to study the mechanisms of PDA coating and the immobilization of growth factors to PDA for improving tissue regeneration. Most of the current research on PDA-modified scaffolds and their impact on tissue regeneration is conducted through *in-vitro* and *in-vivo* experiments, many experimental outcomes have confirm that PDA plays a significant role in this domain. There is a need for further research to better understand the underlying mechanisms of PDA-cell interactions and their functions *in-vivo*, as well as to identify the requirements for future experiments to clarify the role of PDA in clinical practice.
